# YY1-Binding Sites Provide Central Switch Functions in the PARP-1 Gene Expression Network

**DOI:** 10.1371/journal.pone.0044125

**Published:** 2012-08-28

**Authors:** Martina Doetsch, Angela Gluch, Goran Poznanović, Juergen Bode, Melita Vidaković

**Affiliations:** 1 Helmholtz Centre for Infection Research/Epigenetic Regulation, Braunschweig, Germany; 2 Department of Biochemistry and Molecular Cell Biology, Max F. Perutz Laboratories, University of Vienna, Vienna, Austria; 3 BIOBASE GmbH, Wolfenbuettel, Germany; 4 Department of Molecular Biology, Institute for Biological Research, University of Belgrade, Belgrade, Serbia; 5 Hannover Medical School (MHH), Experimental Hematology, Hannover, Germany; University of Massachusetts Medical, United States of America

## Abstract

Evidence is presented for the involvement of the interplay between transcription factor Yin Yang 1 (YY1) and poly(ADP-ribose) polymerase-1 (PARP-1) in the regulation of mouse PARP-1 gene (*muPARP-1*) promoter activity. We identified potential YY1 binding motifs (BM) at seven positions in the *muPARP-1* core-promoter (−574/+200). Binding of YY1 was observed by the electrophoretic supershift assay using anti-YY1 antibody and linearized or supercoiled forms of plasmids bearing the core promoter, as well as with 30 bp oligonucleotide probes containing the individual YY1 binding motifs and four *muPARP-1* promoter fragments. We detected YY1 binding to BM1 (−587/−558), BM4 (−348/−319) and a very prominent association with BM7 (+86/+115). Inspection of BM7 reveals overlap of the *muPARP-1* translation start site with the Kozak sequence and YY1 and PARP-1 recognition sites. Site-directed mutagenesis of the YY1 and PARP-1 core motifs eliminated protein binding and showed that YY1 mediates PARP-1 binding next to the Kozak sequence. Transfection experiments with a reporter gene under the control of the *muPARP-1* promoter revealed that YY1 binding to BM1 and BM4 independently repressed the promoter. Mutations at these sites prevented YY1 binding, allowing for increased reporter gene activity. In PARP-1 knockout cells subjected to PARP-1 overexpression, effects similar to YY1 became apparent; over expression of YY1 and PARP-1 revealed their synergistic action. Together with our previous findings these results expand the PARP-1 autoregulatory loop principle by YY1 actions, implying rigid limitation of *muPARP-1* expression. The joint actions of PARP-1 and YY1 emerge as important contributions to cell homeostasis.

## Introduction

Poly(ADP-ribose) polymerase-1 (PARP-1) is the principal member of the PARP family of enzymes that utilize β-NAD^+^ as a substrate to synthesize and transfer ADP-ribose polymers to acceptor proteins, including itself (automodification). PARP-1 was initially identified as a central component of the DNA repair pathway for single-stranded breaks. For some time its enzymatic activity was thought to strictly depend on its association with free DNA ends which increases its activity 10–500 fold due to allosteric actions. Subsequent studies have expanded the list of its functions and have led to the conclusion that PARP-1 is a constitutively-expressed, multifunctional enzyme for which DNA damage-induced hyper activation is just one out of several options [1], [2]. In addition to its function as a DNA-damage sensor, the enzyme contributes to DNA methylation and imprinting [3], insulator activity [4], chromosome organization [5], the regulation of telomere length [6] and aging [7], [8]. PARP-1 is also involved in transcription regulation [9] and acts as an important modulator of transcriptional processes, enabling cells to cope with noxious stimuli [10].

It is now firmly established that PARP-1 responses to extreme stress stimuli may lead to cytotoxic over-activation *via* the DNA damage-induced route [1], [11]. According to current view, PARP-1 is a well known apoptotic marker [12]. Its hyperactivity depletes the energy-donor molecules NAD^+^ and ATP, which in turn induces necrotic pathways. A contribution of PARP-1 to cell death by mediating translocation of apoptosis-inducing factor (AIF) from the mitochondria to the nucleus has also been found [13]. These and other related findings implicate PARP-1 in many aspects of cell survival. At present, PARP-1 is considered as a molecular switch which affects cell homeostasis and the choice of cell death pathways [1], [14]. Its contribution to systemic pathophysiological phenomena is recognized and has major implications for human health, disease [1], [15]–[17] and response to anticancer therapy [18], [19]. Not all disorders related to PARP-1 can be ascribed, however, to its over-activation since low activities have been mentioned in the etiology of reduced pro-inflammatory mediators, tissue damage and in reperfusion injury [20]–[22]. Together, these findings reveal the intricate balance of the cellular responses that modulate PARP-1 activity [23], [24]. While PARP-1 inhibitors emerge as novel therapeutic tools to limit cellular injury and inflammation and to enhance the efficacy of anticancer therapies [15], [16], [25]–[28], we have yet to refine our understanding of the pathways that determine its enzymatic activity and the molecular details that control its expression. It is expected that only deeper knowledge about the modes of PARP-1 regulation will enable novel therapeutic regimens.

To date the promoters of PARP-1 genes in humans [28], rats [29] and mice [30], [31] have been cloned, and relevant binding sites for transcription factors Sp1, AP-2 [30], YY1 [32], Ets [33] and NF1 [34] determined. Recent sequencing efforts led to a further expansion of this list [35] by revealing binding sites for multiple candidate regulatory factors in the distal region of the human PARP-1 promoter, such as: CDE, GKLF, BARB, MAZF, RREB, HOX, GSH-1, CEBP*β*, E4BP4, STAT6, cETSZ-1, Pbx-1, TCF/LEF-1, NF-*κ*B, c-Rel, ZBP-89, SP-1, CPBP, MAZF, USF, CDF-1, EGR-1, Egr-1/Krox-24/NGFI-A and Ikaros 1.

Our present attempts to advance knowledge of muPARP-1 gene (*muPARP-1*) transcription regulation have focused on the ubiquitous zinc finger transcription factor Yin Yang 1 (YY1) [36]. YY1 plays important roles in the regulation of many genes involved in a variety of cellular functions and biological processes responsible for maintaining cellular stability and physiology [37]–[39]. Acting as either a transcriptional repressor or activator, YY1 has the ability to initiate and regulate transcription depending on the physiological and cellular context [40]. The initial identification of YY1 as a DNA-binding nuclear matrix protein (originally called “NMP1”) [41] was related to a regulatory element next to the histone H4 gene. Subsequent identification of YY1 consensus sequences adjacent to DNA unpairing elements (UEs) [36] confirmed that, in this setting, YY1 mediates gene-nuclear matrix interactions [1]. These and other observations [42] suggest that YY1 participates in the assembly of multi-molecular gene-regulatory complexes containing PARP-1 that are modulated in a dynamic fashion by auxiliary proteins [43]. Indirect support for this comes from the observation that immediately after genotoxic treatment of HeLa cells, YY1 associates with the BRCT motif in the PARP-1 automodification domain [44] to accelerate DNA repair [45]. Subsequent transient poly(ADP-ribosyl)ation of YY1 [46] reduces its DNA binding affinity. Functional relations between YY1 and PARP-1 are also relevant in cases where enzymatic PARP-1 activity modulates transcription. Recently detected gender differences confirm the contribution of exogenous factors to PARP-1 regulation [47]. These findings lend further support to the view that, like YY1, PARP-1 acts in a context-dependent manner, exerting either activating or repressing effects.

Results from several laboratories [31], [48], [49] have provided evidence that PARP-1 gene expression is controlled by an autoregulatory loop in which the enzyme suppresses its own promoter. The central components of this negative-feedback mechanism have been identified: a proximal scaffold/matrix-attachment region (S/MAR) that acts as an upstream control element in conjunction with the *muPARP-1* promoter, and a novel consensus motif (AGGCC) which mediates PARP-1 binding to three sites within the promoter [31]. Information, according to which the *muPARP-1* promoter contains YY1 recognition sequences in the immediate upstream region, has motivated our present study in which these motifs were subjected to a critical evaluation by testing their influence on promoter activity. To this end, we first examined the binding of YY1 to these sites both *in vitro* and *in vivo.* Subsequent transfection studies and mutation experiments revealed major effects of three identified binding sites on the *in vivo* expression of a luciferase reporter gene. While YY1 dampens reporter gene activity by associating with two of these sites, its expression was restored by their mutation. Our findings provide strong evidence that YY1 has the capability to down regulate the PARP-1 promoter. These results are combined in a working model in which YY1 supports the PARP-1 auto-regulatory loop to enable a variety of reduced expression levels. As these actions may serve to restrict and tune energy consumption, YY1 appears as an important contributor to the energy balance within a cell [1].

## Results

### Identification of YY1 Binding Sites in the 774 bp *muPARP-1* Minimal Promoter

**Figure 1:**
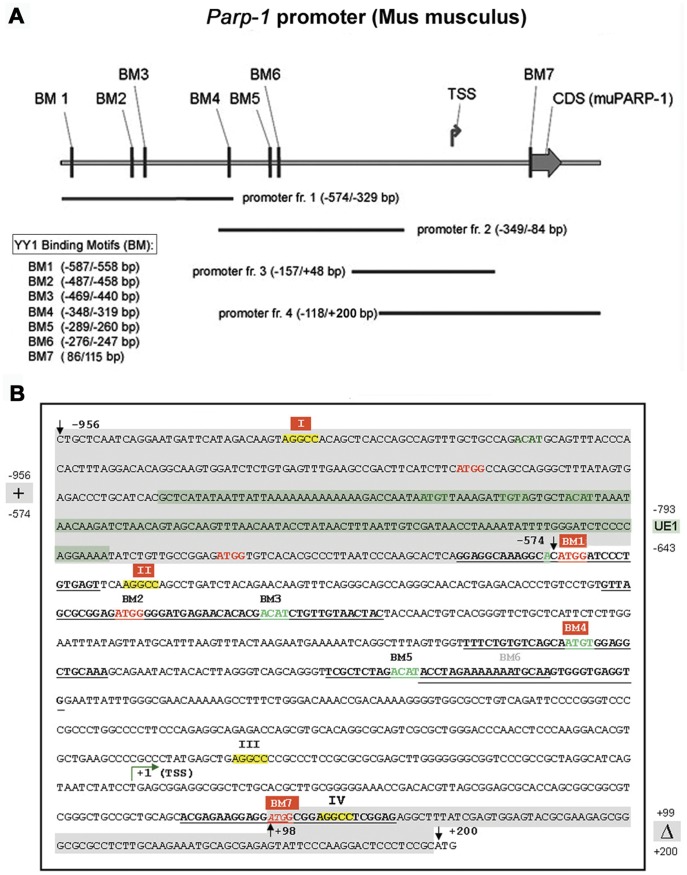
The mouse minimal PARP-1 gene promoter, its binding motifs and extensions. (A) The *muPARP-1* core promoter as predicted by *Genomatix* (−572/+202 bp) as described previously [31]. The 30 bp long oligonucleotides (BM 1 to 7) contain potential YY1 binding motifs and a negative control (BM6). Fragments 1 to 4 cover the entire promoter range with some overlaps (evaluated in Fig. 4). TSS – transcription start site (position +1); CDS – coding sequence. (B) Localization of YY1 biding motifs (BM1-7). The representation covers PARP-1 promoter upstream extension containing the functional PARP-1 binding motifs AGGCC (I), (highlighted in yellow and labelled with Roman numerals). The examined consensus PARP-1 [31] or YY1 sequences (in this paper) are framed by the red rectangles.

This study explores the transcriptional regulation of the *muPARP-1* promoter by YY1 and extends our earlier work which dealt with an autoregulatory loop by which PARP-1 can limit its own expression [31]. Initial professional analyses (Genomatix Software GmbH, Munich) predicted the *muPARP-1* minimal promoter to extend over 774 bp (positions +200 to −574; Fig. 1). In this range, six prototype YY1 core motifs (‘CCAT/ATGG’ or ‘ACAT/ATGT’) [50] were identified at seven positions (BM1 to BM7; Fig. 1A). YY1 binding to the *muPARP-1* core promoter was subsequently examined in electrophoretic mobility shift assay (EMSA) experiments.

**Figure 2:**
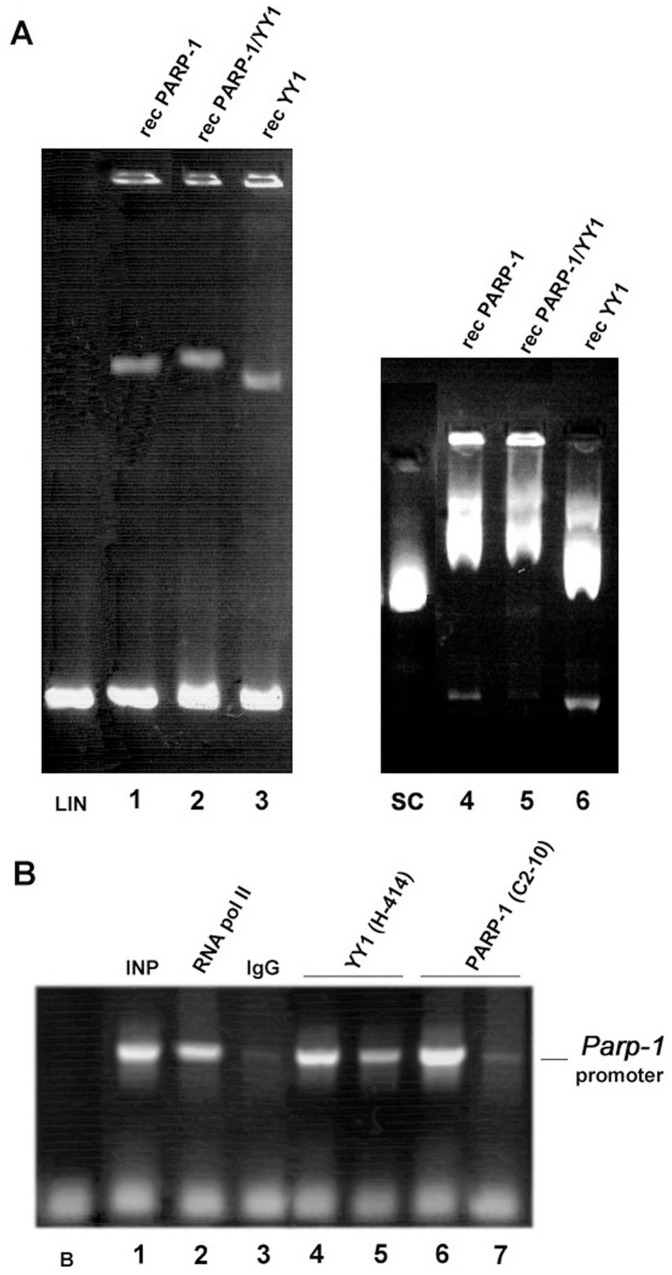
YY1 binding affinity for the *muPARP-1* core promoter. (A) EMSA was performed with either the linearized or a circular, supercoiled 774 bp PARP-1 minimal promoter segment as part of the pSLGTKneo vector backbone. The assay involves incubation with recombinant PARP-1 protein or YY1 protein alone, or with both proteins at a 1∶1 molar ratio. Analyses are performed on non-denaturating 1% agarose gels. Complex formation for the linearized *muPARP-1* promoter fragment (“LIN”) and the vector-containing PARP-1 promoter (“SC”), was visualized with ethidium bromide. (B) The *in vivo* binding affinity of YY1 towards the PARP-1 promoter was confirmed by ChIP analysis with anti-YY1 antibody (H-414, Santa Cruz) as indicated. PARP-1 binding served as a positive control [31]. The anti-PARP-1 antibody was C2-10 from Alexis. Lane B – blank; no DNA template; lane 1– input DNA; 2– RNA pol II, positive control antibody; 3– IgG, negative control antibody; lane 4– NIH3T3 cell chromatin pull-down with YY1 antibody; 5– PARP^−/−^ cell chromatin pull-down with YY1 antibody; 6– NIH3T3 cell chromatin pull-down with PARP-1 antibody; 7– PARP^−/−^ cell chromatin pull-down with PARP-1 antibody.

Non-radioactive EMSA was first performed using the minimal promoter segment in its linearized and supercoiled forms (this template was obtained by cloning *muPARP-1* into the pSLGTKneo vector backbone) (Fig. 2). Due to its strand-separating propensity, the covalently-closed circular (supercoiled; SC) variant might be expected to better reflect the promoter’s native status. Besides, it safely circumvents contributions caused by association of the relevant factors with free DNA ends. Both structural variants were incubated with either PARP-1 or YY1 alone or were provided with both proteins at a 1∶1 ratio. Results in Fig. 2A reveal protein-*muPARP-1* DNA binding between recombinant PARP-1 protein (lanes 1 and 4) and YY1 protein (lanes 3 and 6), to the linearized (lanes LIN and 1–3) and supercoiled promoters (lanes SC and 4–6). Mutual interactions of PARP-1 and YY1 within the *muPARP-1* promoter are reflected by the nucleoprotein complex derived from PARP-1 *plus* YY1 (lanes 2 and 5: linearized and supercoiled *muPARP-1* DNA, respectively) that migrates more slowly than the respective nucleoprotein complexes for PARP-1 or YY1 alone.

In order to confirm YY1 binding to the PARP-1 promoter *in vivo*, we performed chromatin immunoprecipitation (ChiP) experiments with NIH3T3 wt (PARP-1^+/+^) and PARP-1 knock-out (PARP-1^−/−^) fibroblasts (Fig. 2B). Cis-DDP is the preferred crosslinking agent since it introduces reversible protein-DNA links in the absence of protein-protein links, which could affect the results [31]. DNA was released from the nucleoprotein complexes by adjusting the concentration of Cl^−^ ions, purified and analyzed by PCR using primers flanking *muPARP-1* promoter. Results of the ChIP experiments with anti-YY1 antibody reveal the *in vivo* binding affinity of YY1 for the *muPARP-1* promoter in both PARP-1^+/+^ (lane 4) and PARP-1^−/−^ (lane 5) cells.

### Affinity of YY1 for Six Prototype Binding Motifs in the *muPARP-1* Promoter

**Figure 3:**
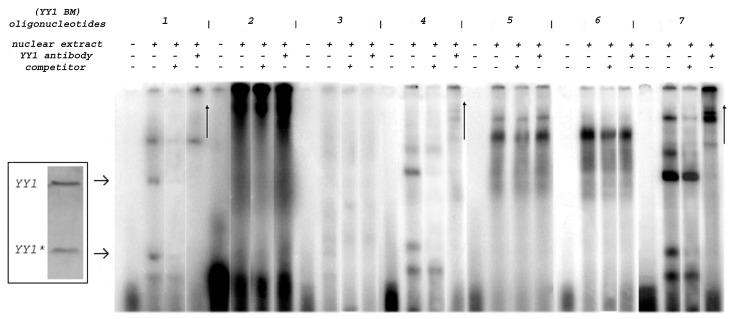
Differences in the avidity of YY1 association examined for six potential binding motifs in *muPARP-1*. Oligonucleotides containing the YY1 binding motif (BM1 to 5, 7) and a control (BM6) served as probes for EMSA. For each radioactive probe the binding reaction was performed either in the absence or presence of NIH3T3 nuclear extract. A third competition reactions contained a 200-fold molar excess of particular unlabeled oligonucleotides (BM 1–7) in order to illustrate the specificity of the protein:DNA interactions. A fourth reaction contained anti-YY1 antibody (H-414; Santa Cruz). Samples were run on an 8% polyacrylamide gel. Arrows indicate bands shifted by YY1/oligonucleotide binding alone (central lines in each group) whereas supershifts by the antibody are evident in the rightmost lanes for BM1, BM4 and BM7. Inset – Immunoblot analysis of NIH3T3 cell lysates revealed the presence of a degradation product (YY1*), as already reported [52].

Having established the *in vivo* association of YY1 with the *muPARP-1* promoter, we assessed differences in YY1 binding to the motifs identified *in silico* (Fig. 1). Seven 30 bp oligonucleotide probes (containing motifs BM1 to 7) were prepared and subjected to EMSA. For each radioactive probe, binding reactions were performed in the absence or in the presence of a NIH3T3 nuclear extract; a third reaction contained appropriate unlabeled oligonucleotide (BM 1–7 respectively) as competitor, and the fourth reaction contained an anti-YY1 antibody (Fig. 3). All seven oligonucleotide probes gave raise to multiple bands (Fig. 3). Specificity of protein:DNA binding was confirmed using unlabeled oligonucleotides as a competitor. YY1 binding was documented by super-shifts in the presence of YY1 antibody (rightmost lane in each of groups 1–7). These reveal binding of either YY1 alone or of YY1 as a part of protein complexes. A degradation product of YY1 in the NIH3T3 nuclear extracts appeared in the immunoblots as observed previously [51], indicating that some of the bands may represent complexes between the degradation product and the oligonucleotide probes (“YY1*” in the Fig. 3 inset). Other bands (not considered in Fig. 3) resulted from DNA binding proteins other than YY1. These data show that of the analyzed DNA probes, the YY1 binding motifs contained in BM1 (−587/−558 bp), BM4 (−348/−319 bp) and BM7 (+86/+115 bp) associated with YY1, and that the most pronounced binding was displayed by BM7.

### YY1 Association with Overlapping Sections of the *muPARP-1* Minimal Promoter

**Figure 4:**
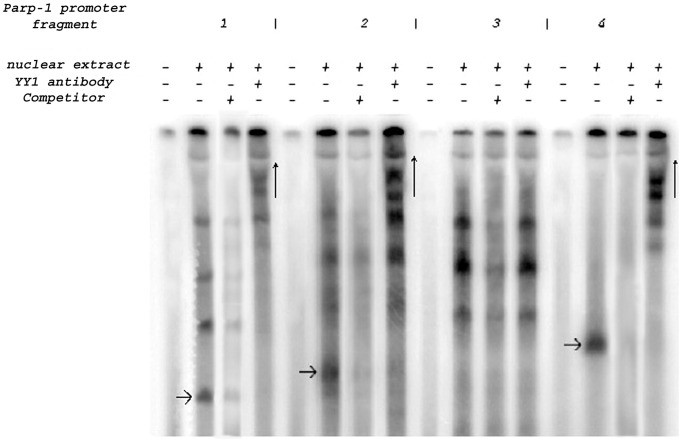
Additional proof for YY1 binding obtained using *muPARP-1* promoter fragments for EMSA. YY1 binding to the three motifs BM1, BM4 and BM7 within the *muPARP-1* promoter fragments (“promoter fragments 1–4” in Fig. 1A) was confirmed by EMSA. Radioactively labelled probes were incubated with or without nuclear extract. A third competition reactions contained a 200-fold molar excess of particular unlabeled *muPARP-1* promoter fragments 1–4 in order to illustrate the specificity of the protein:DNA interactions. A fourth binding reaction contained anti-YY1 antibody. Samples were run on a 5% polyacrylamide gel. Arrows indicate the YY1-probe complexes that were supershifted.

In order to examine the extent to which YY1 association with the restricted binding motifs depends on cooperative interactions between these sites, the PARP-1 promoter was divided into four more extended, overlapping sections designated as promoter fragments (fr.) 1–4 (Fig. 1A). These fragments were amplified by PCR, cloned into pCR2.1 TOPO, cut from the vector backbone and radioactively labelled. They were incubated either with or without the NIH3T3 nuclear extract; a third binding reaction contained appropriate unlabeled *muPARP-1* promoter fragments (1–4, respectively) as competitor; the fourth reaction contained the YY1 antibody (Fig. 4). The occurrence of nucleoprotein complexes in the samples containing fragments 1, 2 and 4, nuclear extracts and antibody (vertical arrows) prove YY1 binding to fragments 1, 2 and 4 but not to fragment 3. This was expected in view of the previous results of YY1 binding to the 30 bp probes BM1, BM4 and BM7 (Fig. 3). This result indicates that the binding of YY1 to promoter fr. 1 was mediated by BM1 (−587/−558 bp) and BM4 (−348/−319 bp), whereas YY1 binding to promoter fr. 2 was mediated by BM4 which resides in a region contained in both promoter fr. 1 (−574/−329 bp) and promoter fr. 2 (−349/−84 bp), respectively). In an analogous manner, YY1 binding to promoter fr. 4 (−118/+200 bp) was mediated by the BM7 motif (+86/+115 bp).

### The Kozak Sequence is the Central YY1 Binding Region in the *muPARP-1* Promoter

The results presented in Fig. 4 show that the promoter fr. 4 (the section comprising the YY1 motif BM7) forms the most pronounced complex, which supports and amends the results in Fig. 3. The presentation of the core *muPARP-1* promoter in Fig. 1 A/B shows that BM7 resides downstream from the *muPARP-1* transcription start site (TSS). Inspection of its sequence (Fig. 1B) reveals an overlap of the *muPARP-1* translation start site (5′-GG AGG *ATG* GCG GAG-3′ at position +93/+115 bp), the Kozak consensus sequence [(5′-gcc)gccA/GccATGG-3′], the YY1 ‘*ATG* G’ core, and the PARP-1 consensus sequence (AGGCC). This observation agrees with the co-localization of the YY1 motif and the translation start site in many human promoters [51]. Together these results confirm that the high-affinity YY1 site, BM7, contains or supplements sequences with potential relevance for *muPARP-1* promoter function.

**Figure 5:**
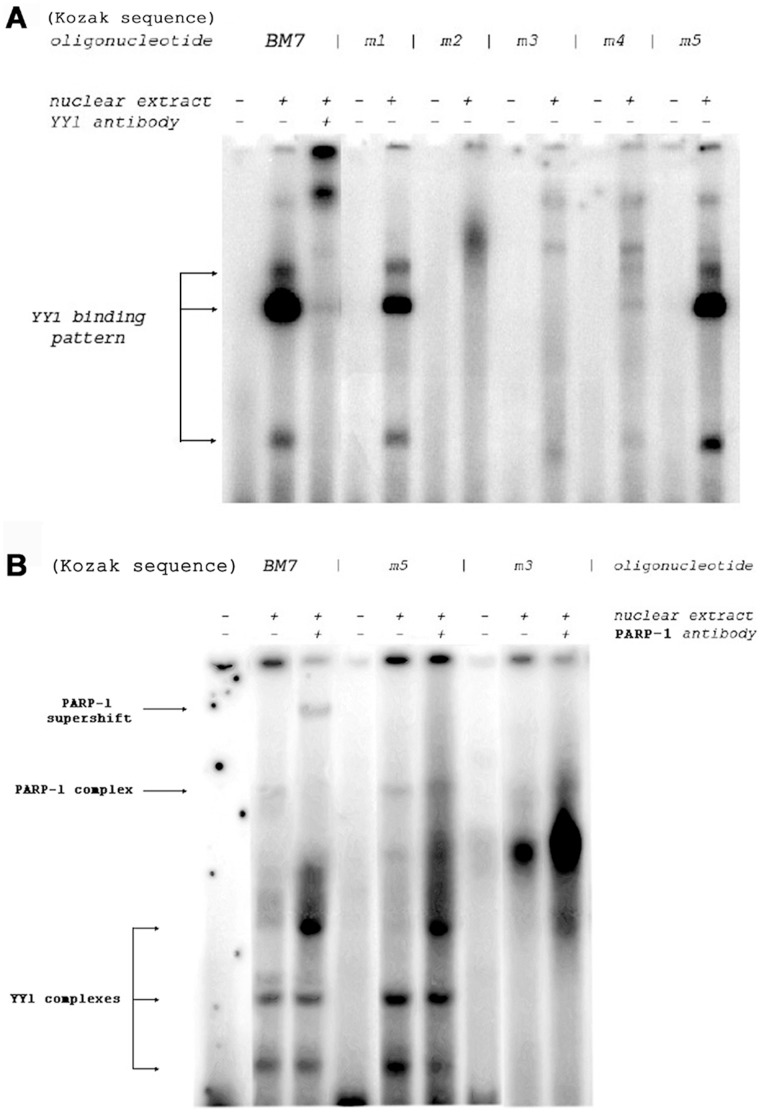
YY1 binds the Kozak sequence as the most prominent binding motif and assists PARP-1 binding. (A) Mutation of the YY1 core sequence within BM7 abolished YY1 binding as shown by super shift experiments with anti-YY1 antibody. (B) EMSA experiments performed with anti-PARP-1 antibody and selected mutated oligonucleotides m3 and m5 revealed that YY1 protein is required for PARP-1 binding to its consensus sequence located next to the Kozak sequence. The sequences of the double stranded oligonucleotides used as probes are as follows (small letters indicate the mutated positions): wildtype (BM7) 5′ ACG AGA AGG **A**GG **ATG G**CG GAG GCC TCG GAG 3′ mutation 1 (m1) 5′ ACG Atc ctt **A** GG **ATG G**CG GAG GCC TCG GAG 3′ mutation 2 (m2) 5′ ACG AGA AGt ctt cTG GCG GAG GCC TCG GAG 3′ mutation 3 (m3) 5′ ACG AGA AGG AGG cgt taG GAG GCC TCG GAG 3′ mutation 4 (m4) 5′ ACG AGA AGG **A**GG **ATG G**at tct GCC TCG GAG 3′ mutation 5 (m5) 5′ ACG AGA AGG **A**GG **ATG G**CG GAt taa gaG GAG 3′. Each probe (referred to as m1 to m5) was incubated in the absence or the presence of nuclear extract and examined by EMSA. Wild type BM7 was also incubated with nuclear extract and antibody to identify the bands that are shifted by YY1 or PARP-1 binding. Samples were run on a 8% polyacrylamide gel. The Kozak consensus sequence (gcc)gccRccATGG for which R is a purine three bases upstream of the start codon (AUG), is followed by another ‘G’, and is in bold capital letters.

These data motivated efforts to characterize the interactions of BM7 with YY1 and with PARP-1. First, BM7-YY1 binding was analysed in detail by mutating BM7 and the surrounding nucleotides. Mutations (“m1” through “m5”) covering 5 base pairs within the BM7 wild type sequence 5′-GA AGG AGG ATG GCG GAG-3′ were created by site-directed mutagenesis and used for the EMSA experiments presented in Fig. 5A. The three bands found to be associated with YY1 binding to wild type BM7 were likewise present for mutants m1 and m5 (vertical arrows). The absence of these bands for m2 and m3 (which contained altered YY1 cores and Kozak motifs), confirmed that YY1 binding was abolished. Compared to the three bands in native BM7, the bands for m4 are vanishingly weak. This result proved that the nucleotide bases directly downstream from the core motif ‘ATGG’ contributed to the YY1 binding. In another set of EMSA experiments in which BM7-PARP-1 binding was examined (Fig. 5B), we used the wt BM7 sequence and the following two mutated oligonucleotides: m3 (containing the mutated YY1 core binding motif; ATGG  = cgtt), and m5 (with the mutated PARP-1 consensus sequence; AGGCC  = Attaaga). The super shift obtained with anti-PARP-1 antibody revealed PARP-1 binding to wt MB7, presumably to its AGGCC consensus sequence. Also, in the same reaction a common YY1 binding pattern to BM7 oligonucleotide was detected. No PARP-1 binding was detected after supershift analysis with the m5 oligonucleotide containing mutated AGGCC, while YY1 was able to bind mutated m5 oligonucleotide since its core binding motif was intact (Fig. 5A, B). When EMSA was performed with the m3 oligonucleotide probe with the mutated core YY1 binding motif (ATGG) and the unchanged PARP-1 consensus sequence, no YY1 and no PARP-1 binding was detected. This experiment revealed that PARP-1 binding is absolutely dependent on the presence of YY1 protein when the PARP-1 consensus sequence is located next to the YY1 binding motif in the *muPARP-1* gene promoter.

So far our findings provide clear evidence that BM7 comprises an YY1- as well as a PARP-1 binding region within the Kozak sequences. Extending our interpretation we can also conclude that the Kozak consensus sequence located in BM7 supports the association of YY1 with the *muPARP-1* promoter and that the presence of YY1 protein is indispensable for PARP-1 binding.

### Functional Consequences of YY1 Binding to the *muPARP-1* Promoter: Effects on Reporter Gene Expression

**Figure 6:**
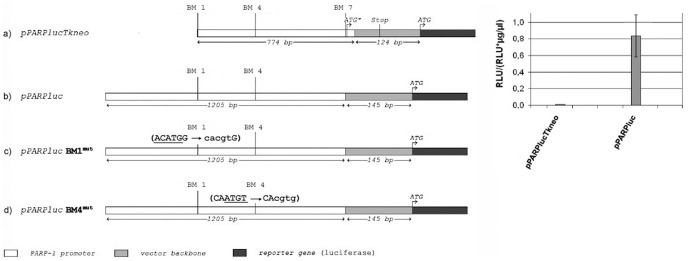
Essential *muPARP-1* promoter regions identified in reporter plasmids. Reporter plasmids were pPARPlucTkneo, pPARPluc, pPARPluc**BM1^mut^** (mutated YY1-binding motif in binding motif BM1) and pPARPluc**BM4^mut^** (mutated YY1-binding motif in BM4). The YY1-binding motifs BM1, BM4 and BM7, the reporter gene translation start codon (ATG), the PARP-1 translation start codon (ATG*) and the stop codons that follow the PARP-1 gene translation start are indicated. The *muPARP-1* core-promoter predicted by *Genomatix* is contained in pPARPlucTkneo. To provide expression levels sufficient for the evaluation of PARP-1 promoter functions, the sequence must be extended upstream, but it has to exclude the translation start codon, the overlapping YY1-binding motif in BM7 and a minor part of the PARP-1 coding sequence. These changes permit analyses based on the luciferase (luc-) reporter as demonstrated in the inset. The corresponding analyses on mutants m1′ (ACATGG → cacgtG) and m2′ (CAATGT → CAcgtg) are applied to confirm increase of *muPARP-1* promoter activity relative to the wt sequences (Fig. 8).

The next level of our study addressed the functional consequences of YY1 binding to the *muPARP-1* promoter. To this end, the 774 bp *muPARP-1* core promoter (Fig. 6) was cloned into a luciferase/green fluorescent protein (GFP) fusion gene expression vector (pPARPlucTkneo), which was used, in turn, to transfect NIH3T3 cells. As this reporter plasmid showed limited activity it had to be adjusted for further use.

We have previously shown that a reporter plasmid containing the *muPARP-1* core promoter with a 384 bp extension at its 5′ end and a slightly reduced 3′ end (deletion “Δ +99 – +200” in Fig. 1B), provides considerable transcriptional potential [31]. Plasmid pPARPluc comprises 1054 bp, i.e. the region between positions −956 and 98 bp (Fig. 1B; schematic representation in Fig. 6). The 5′ extension contains an unstable DNA base-unpairing element, designated “UE1” in Fig. 1B (cf. Fig. 6) [31] between positions −793 and −643; this sequence provides an enhancer-like effect and poses the YY1-binding motifs BM1 (−587/−558 bp) and BM4 (−348/−319 bp) into a downstream position (Fig. 1B). Since our study was focused on deactivating contributions, the molecular basis of the UE1-dependent enhancement exceeded our present topic and the element was applied as an unaltered building block. In addition to this change, the 3′ end of the PARP-1 promoter in pPARPluc had to be trimmed, whereby the BM7 motif, the overlapping Kozak sequence and a minor part of the PARP-1 coding sequence (Fig. 1B) were removed. As all following investigations relied on the improved expression activities of the *pPARPluc* relative to *pPARPlucTkneo* (inset to Fig. 6), we had to refrain from a further characterization of the strong YY1 site BM7.

**Figure 7:**
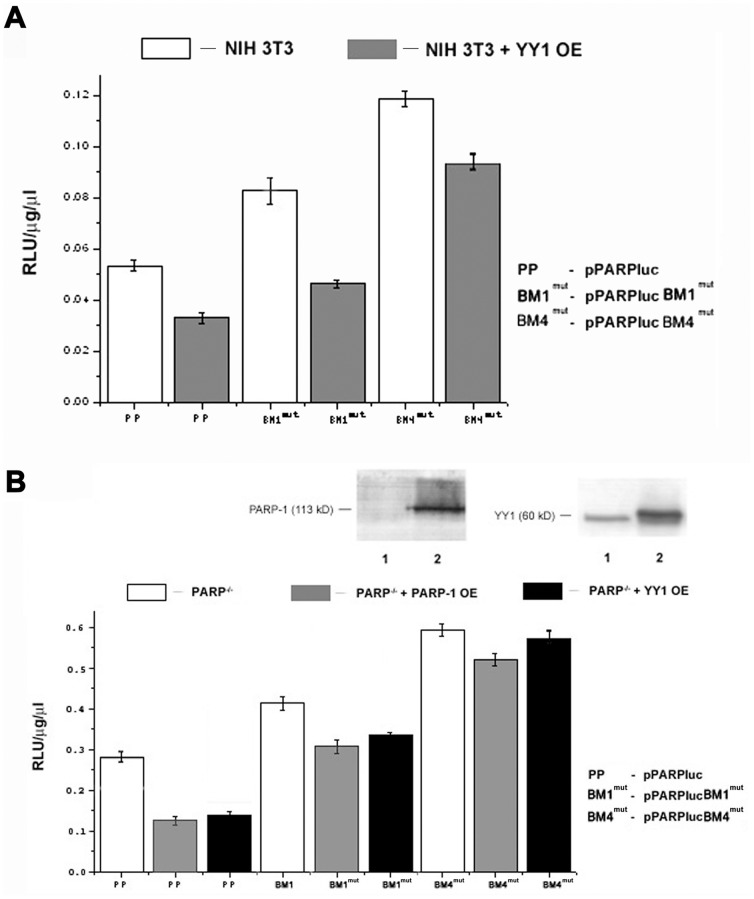
PARP-1 and YY1 downregulate *muPARP-1* promoter activity. (A) YY1 downregulates *muPARP-1* promoter activity. Comparison of the transcriptional activities of the wt, pPARPluc luciferase reporter driven by the extended PARP-1 promoter (PP) and reporter gene constructs pPARPlucBM1^mut^ (**BM1^mut^**) and pPARPlucBM4^mut^ (**BM4^mut^**) containing mutated YY1-binding core motifs BM1 and BM4, respectively as indicated in Fig. 7. To test transfection efficiencies, NIH3T3 cells were co-transfected with pMDICluc. For YY1 overexpression (grey bars), cells were co-transfected with pcDNA3.1FLAGYY1. Firefly luciferase activities of the reporter vectors are normalized to *Renilla* luciferase activity of the control plasmid pMDICluc and to the protein concentration. YY1 overexpression was confirmed by immunoblot analysis (figure inset); lane 1– NIH3T3 cell lysate; lane 2– NIH3T3 cell lysate after pcDNA3.1FLAGYY1 transfection. OE – overexpression. (B) PARP-1 and YY1 downregulate *muPARP-1* promoter activity. Transfection experiments using a luciferase assay were performed in PARP-1 knockout NIH3T3 cells (PARP^−/−^). The reporter (*pPARPluc* and its mutants **BM1^mut^** and **BM4^mut^**) have been introduced in Fig. 7. For PARP-1 overexpression (light grey bars), cells were co-transfected with pECVPARP, which is a PARP-1 cDNA expression construct; for YY1 overexpression (dark bars), pcDNA3.1FLAGYY1 was used. Overexpression of PARP-1 and YY1 was again confirmed by immunoblot analysis; lane 1– PARP^−/−^ NIH3T3 cell lysate; lane 2– PARP^−/−^ NIH3T3 cell lysate after co-transfection with pECVPARP or pcDNA3.1FLAGYY1, as indicated. OE – overexpression.

For present purposes, we created mutant reporter gene constructs (pPARPlucBM1^mut^  =  BM1^mut^ and pPARPlucBM4^mut^  =  BM4^mut^), for which the YY1 core motif ACATGG was either converted to cacgtG, (BM1^mut^) or CAATGT to CAcgtg (BM4^mut^; cf. Fig. 7) [31], to be used for expression studies in NIH3T3 cells. Fig. 7A shows that the mean value for the wild type plasmid (PP) was significantly lower than for the plasmid with a BM1 mutant site (pPARlucBM1^mut^) and also for the plasmid with a mutation in BM4 (pPARPlucBM4^mut^; white bars). These results also show that mutations at sites BM1 and BM4 affected YY1 association, providing higher reporter gene expression compared to the wild type reporter plasmid pPARPluc.

Further use of reporter gene constructs was made to explore the contribution of YY1 to PARP-1 promoter function under conditions of YY1 overexpression (Fig. 7A, filled grey bars). These conditions were established by transfection with a vector, which included a human YY1 expression unit (pcDNA3.1FLAGYY1). We expected that increased YY1 levels would lead to further repression relative to the physiological state. Since both the murine and human YY1 gene open reading frames show 94.9% sequence similarity [40], it could be anticipated that the human YY1 protein introduced into a mouse cell possessed properties and functions comparable to its murine counterpart. YY1 overexpression was verified two days after transfection by immunoblot analysis of cell lysates with anti-YY1 (inset to Fig. 7A). The band corresponding to murine YY1 in the control (insert, lane 1) was also present in the lysate prepared from cells over-expressing YY1 (lane 2), which is documented by the dominant band (FLAG-tagged huYY1) slightly above the murine YY1 signal. These analyses confirmed that the levels of human YY1 greatly exceeded those of the intrinsic murine gene. Comparing reporter expression at endogenous levels of YY1 (“NIH3T3”) relative to those obtained under conditions of overexpression (“NIH3T3+ YY1 OE”) confirmed that luciferase activity was reduced by approximately one third (Fig. 7A). Comparison with promoters mutated at BM1 and BM4 (BM1^mut^and BM4^mut^) verified that the interactions of YY1 with these sites were responsible for *muPARP-1* promoter down regulation. These results also proved that the over-expression of YY1 was inadequate to dampen gene expression to the extent observed for wild type BM1 and BM4.

So far, this study was focused on YY1-mediated regulatory mechanisms that control *muPARP-1* promoter activity. With the knowledge that elevated PARP-1 levels suppress PARP-1 expression at the transcriptional level [31], we examined whether down-regulation by both factors (PARP-1 and YY1) can occur independently, in an additive or in a synergistic fashion. To this end, PARP^−/−^ cells were transfected with either the luciferase wild type reporter plasmid (pPARPluc; left triplet of bars), or with one of the two constructs containing mutated BM1 (center triplet with BM1^mut^ marks) or BM4 sites (right triplet carrying BM4^mut^ marks) as noted above. In general agreement with the results presented in Fig. 7A, Fig. 7B shows that the BM1 and BM4 mutants relieved the repressive actions of YY1 and notably also of PARP-1 (white bars). The latter effect could indicate indirect, i.e. remote interactions of PARP-1, at least with these YY1 binding sites.

Additional transfection of PARP^−/−^ cells with either a PARP-1 (gray bars) or with the YY1-overexpression construct (black bars) decreased reporter gene activity for all constructs, i.e. for the native BM1/BM4 promoters as well as for its mutants (BM1^mut^/BM4^mut^). In cells that carried the mutant sites a general recovery of reporter gene activity was noted. At the same time the differences relative to the unmodified situation disappeared. In all cases, PARP-1 overexpression (gray bars) reduced reporter gene activity somewhat more than YY1 (black).

In conclusion, the results presented in Fig. 7A and B present unequivocal evidence that the upstream YY1 binding sites BM1 and BM4 are responsible for the down regulation of the *muPARP-1* promoter by YY1 (black relative to white bars), and (indirectly) also by PARP-1, at least in the presence of endogenous levels of YY1 (grey relative to white bars). This observation reinforces the conclusion drawn from Fig. 5A and B, that YY1 is required for PARP-1 recruitment to the *muPARP-1* promoter and its DNA binding. Thereby, an additional modulation of *muPARP-1* transcription is enabled by YY1/PARP-1 protein-protein interaction.

## Discussion

This study continues our work on the regulatory mechanisms that down regulate the muPARP-1 gene promoter [31]. We previously derived a model centered on a negative feed-back regulatory loop in which murine PARP-1 gene expression is delimited by the gene product itself. We are now in the position to extend the basic mechanism by considering YY1 interactions with the *muPARP-1* promoter. Several putative YY1 binding sites were predicted *in silico*, out of which binding to three potential sequence motifs (designated “BM1”, “BM4” and “BM7”) could be confirmed by EMSA and ChIP analyses (Fig. 2, 3, 4, 5). The relevance of YY1 interactions with BM1 and BM4 was demonstrated by functional analyses employing transfection and co-transfection procedures, combined with site-directed mutagenesis (Fig. 6 and 7). Our results anticipate corresponding effects in the natural context.

### Activities of the YY1 Upstream Binding Sites BM1 and BM4

In our previous contribution [31] we used the stress-induced duplex destabilization (SIDD) algorithm to predict a distal region of the chromatin domain comprising the muPARP-1 gene and found that its upstream border, “S/MAR 2”, lies between −9500 and about −7500 bp (Fig. 1A/B ibid.). The composite base unpairing structure and its association with lamins A/C is in accord with this function, which in turn supports the action of domain-intrinsic structures such as an unpairing element (UE1). The UE1 is associated with transcription factor binding sites, i.e. PARP-1 and YY1 binding sites (Fig. 1B). The features of UE1 were established by (cis-DDP) crosslinking and functional tests [31], which served to distinguish structure-specific from sequence-specific regulatory functions, the latter being in the focus of the present study.

Three functional YY1 binding sites are distributed across the *muPARP-1* promoter. Whereas BM7 is located downstream (positions +86 to +115 bp), BM4 and BM1 reside upstream at positions −348 to −319 bp and −587 to −558 bp, respectively (Fig. 1B). The distal site, BM1, flanks the mentioned “UE1” element [31] covering positions from −643 to −793 bp. UEs represent distinct sites at which the DNA duplex is strongly destabilized. In SIDD analyses they appear as pronounced minima or destabilized sites [36], [52]–[54]. UEs are related to S/MARs, although the latter consist of an extended series of repetitive, moderately destabilized UEs, which have to comply with a set of well-defined structural rules [55], all of which are met by the mentioned S/MAR 2 element. Since UEs frequently correspond to DNAse I hypersensitive sites with regulatory properties [53], [54], many of these are associated with enhancer-like activities. In the context of our expression vector pPARPluc (Fig. 6), the presence of UE1 (allocated in Fig. 1B between positions −956 and –547 bp) provided the *muPARP-1* promoter with a transcription potential for significant reporter gene expression; in its absence, the activity of the reporter gene was greatly reduced (vector pPARPlucTkneo). Klar and Bode [36] noted that, for the β interferon genes from humans and mice, functional YY1 binding motifs occur at the flanks of destabilized regions. This context is evolutionarily conserved. Being a factor that requires both DNA strands for its binding, YY1 functions may profit from a position next to flexible DNA as some of its actions are associated with its bending potential [56]. Such a situation is found for BM1 and the related UE1-reporter plasmid (pPARPluc; Fig. 6), explaining its pronounced activity relative to pPARPlucTkneo.

The availability of a vector with robust *muPARP-1* promoter activity allowed us to extend the study to functional effects of the distal YY1 binding sites BM 1 and BM4 (Fig. 7). Mutation of these sites (i.e. parts of mutant vectors BM1^mut^ and BM4^mut^, respectively) clearly interfered with YY1 binding. Although YY1 binding to BM4 is more strongly impeded (Fig. 7A), both mutations independently release reporter gene suppression. For NIH3T3 cells lines with either vector mutant, YY1 over-expression could not reduce reporter gene activity to the level observed for the wild-type. This is taken as an indication that, for native BM1 and BM4, suppression is the consequence of site-saturation and it can be anticipated that different degrees of relief from repression will occur at physiological YY1-concentrations. This would imply that dynamic changes of these interactions tune *muPARP-1* promoter activity *in vivo* where these responses follow alterations of environmental stimuli or developmental signals.

Previously we reported that PARP-1 protein at high concentrations exerts a suppressive effect on its own promoter [31]. In the present study, transfection experiments performed in PARP^−/−^ cells (Fig. 7B) add to this information: YY1 overexpression causes >50% suppression of *pPARPluc* (cf. the situation marked “PP”). The presence of mutants BM1^mut^ and BM4^mut^ largely overrides this effect, with the BM4 mutant (BM4^mut^) being the more efficient one. In this case the prominent down-regulation is the result of abrogated YY1/BM4 interactions. While these data support our model in which PARP-1 protein is part of an autoregulatory loop [31], they also show that YY1 represses *muPARP-1* promoter activity by direct interactions with BM1 and BM4. The intriguing finding that PARP-1-mediated suppression under physiological conditions is significantly less pronounced when either of the YY1 binding sites (BM1 or BM4) is mutated indicates that YY1 contributes to this phenomenon in an indirect manner. In summary, PARP-1 and YY1 appear to suppress *muPARP-1* in a synergistic fashion, while YY1 binding to BM1 and BM4 reflect parallel routes of action.

Our expression vector comprises a PARP-1 binding consensus motif (AGGCC) between base pairs −554 and −550 (Fig. 1B, motif “II”, underlined in yellow) adjacent to BM1 (base pairs −587 to −558). Mutations of this tract were shown to prevent PARP-1/promoter interactions and to cause up-regulation of *muPARP-1* [31]. This supports our notion that, at first glance, PARP-1 and YY1 sites are affected separately. Although PARP-1 and YY1 might down-regulate *muPARP-1* independently, it is tempting to speculate that the proximity of BM1 and the PARP-1 site enables protein/protein contacts. This would add yet another level of promoter control involving YY1/PARP-1 crosstalk and it could explain the observation that high PARP-1 levels reduce transcription rates not only by binding to sites I and II, but also by indirect effects due to the BM1 site (Fig. 7B). In this scenario and owing to its DNA-bending potential [56], YY1 binding to BM1 promotes association of PARP-1 with its adjacent binding site “II”. This might allow YY1 to recruit PARP-1 as a corepressor in accord with models by Thomas and Seto [57]. Since PARP-1 and YY1 can enter direct interactions [44], such a crosstalk would determine their mode of binding to DNA [45], [46]. Since the association of YY1 at BM4 causes a greater level of suppression than at BM1, this might represent a dominant switch to control promoter activity. In contrast, association with BM1 could be responsible for chromatin remodeling by PARP-1 to yield a long-lasting but moderate suppression of the promoter according to a previously outlined mechanism [58]. In view of its function as a structural protein, PARP-1 activation induces local conformational changes of chromatin by auto- or hetero-modification. Since there may be a conflict between these effects (chromatin condensation/decondensation), it was suggested that the differential chromosomal distribution of the enzyme permutes locus-specific modulations of chromatin structure [59].

The same type of expression-control could also be valid for the Kozak sequence that carries both the YY1 (BM7 in Fig. 1B) and PARP-1 (IV in Fig. 1B) binding motifs, separated by only 3 bp. The results of EMSA experiments performed with the BM7 oligonucleotide (Fig. 5A and B) provide evidence for a level of *muPARP-1* promoter control *via* YY1/PARP-1 protein-protein interactions [44]. Since the PARP-1 and YY1 binding sites are adjacent, it can be expected that YY1 exerts a pronounced effect on PARP-1 recruitment to the *muPARP-1* promoter, triggering further changes in *muPARP-1* transcription. Our assumption is in accord with the work of Oei and co-workers [32] suggesting that the role of YY1 as a transcriptional cofactor may be tuned by PARP-1 activity.

**Figure 8:**
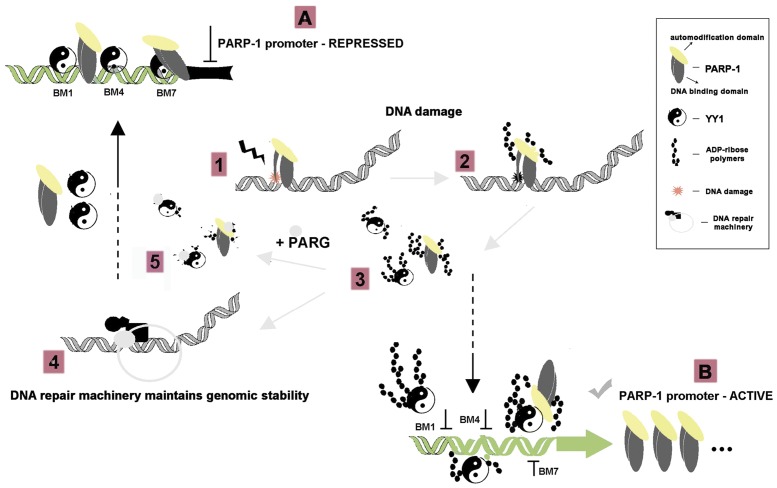
YY1/PARP-1 interplay in *muPARP-1* transcriptional regulation. PARP-1 regulates its own gene transcription by acting as a sequence-specific promoter-binding repressor [31]. Our results suggest that at the basal state, with unmodified PARP-1 binding to DNA and YY1 binding to BM1, BM4 and BM7, *muPARP-1* transcription is maintained at a low physiological level (A). In response to DNA damage anywhere in the genome (1), PARP-1 binds to free DNA ends, which causes a net increase in PARP-1 activity (2). The resulting poly(ADP-ribosyl)ation of free and bound PARP-1 and other target-transcription factors, including YY1, prevents their interaction with the *muPARP-1* promoter (3). Thereby *muPARP-1* is released from the PARP-1/YY1-mediated block and transcription becomes increased (B). In parallel, activated PARP-1 recruits the DNA repair machinery (4). Following DNA repair and removal of poly(ADP-ribose) polymers by poly(ADP-ribose) glycohydrolase (PARG) (5) the DNA binding activity of PARP-1 and YY1 is restored. PARP-1 and YY1, which are stripped of polymers rebind to the *muPARP-1* promoter restoring physiological levels of activity (A).

Under physiological conditions, constitutive binding of YY1 and PARP-1 contribute to the establishment of low levels of *muPARP-1* transcription. In this scenario, interaction between YY1 and PARP-1 is possible if PARP-1 is enzymatically inactive. As certain cellular insults stimulate PARP-1 activity, it is feasible that associations of YY1 with PARP-1 and DNA are tuned by different degrees of poly (ADP-ribosyl)ation. Since this modification releases YY1 from DNA, *muPARP-1* repression will be relieved and muPARP-1 gene expression increased again. Once PARP-1 levels have surpassed a certain threshold, the proposed feedback-type inhibition pathway [31] is initiated. Owing to the comparatively short half-life of poly(ADP-ribose) [57] the regulatory super-cycle is completed by YY1 rebinding. The proposed model is summarized in Fig. 8. It expands on the presumed involvement of YY1 in the regulation of the human PARP-1 promoter [32].

PARP-1 has been implicated in more persistent epigenetic modifications due to its contribution to DNA-methylation patterns, i.e. the inhibitory effect on DNA methyl transferase 1 caused by elevated poly(ADP-ribose) polymer levels [60]. In the same context, we want to emphasize the role of YY1 in limiting PARP-1 activity and point to the possibility that YY1-PARP-1 crosstalk contributes to epigenetic effects. Maintenance of epigenetic actions by YY1-dependent silencing was recently suggested [61]. Acting as a Polycomb group protein (PcG), YY1 recruits chromatin modifiers such as histone deacetylases and histone methyl transferases. The suppressed and principally transient status may be fixed by subsequent DNA methylation [54].

### Potential Role of the BM7 Downstream Region Overlapping the Kozak Sequence

Being located on the *muPARP-1* promoter at a downstream position (+86/+115), the YY1 recognition motif BM7 enables a markedly more stable complex with YY1 than either BM1 or BM4 (Figs. 4, 5, 6, 7). BM7 overlaps the Kozak sequence (positions +89 to +101), which, in vertebrates, determines translation initiation [62]–[66].

YY1 binding to BM7 was proven by site-directed mutagenesis of the respective YY1 core motif (Fig. 5) Our current toolbox did not allow, however, to perform a functional characterization of YY1 binding to BM7 as the “core promoter” had to be trimmed, removing the BM7 motif, the overlapping Kozak sequence and a minor part of the PARP-1 coding sequence. Overlap of BM7 with the consensus Kozak sequence is in accord with data by Xi *et al.* [51] who compiled and analyzed a set of 723 human core promoter sequences for overrepresented motifs. In these cases YY1 motifs mostly reside immediately downstream from the transcription start site (TSS).

It should be pointed out that the location of the TSS in the *muPARP-1* has remained somewhat ambiguous [31]. The promoter is G+C-rich. Lacking a functional consensus TATA box, the *muPARP-1* promoter contains a functional analogue of the TATA box in the form of a 5′-GTAATCT-3′ tract at position −12 to −5. This motif resembles an initiator element (Inr) and synergizes with some upstream binding sites for the strong transcriptional activator Sp1 [31], [67]. Both, TATA box and Inr, provide options for transcription complex formation from Pol II and general transcription factors. The binding of YY1 to the Inr elements of many promoters is well documented [39], [68]–[72]. In this situation, YY1 can assume the role of a transcriptional initiator protein [73], [74]. Following the available information [75], [76], a mechanistic basis for transcriptional initiation directed by YY1 in the absence of the TATA box-binding protein (TBP) emerges, i.e. under appropriate conditions YY1 may take over TBP functions at the Inr element and recruit the large subunit of Pol II.

So far the YY1 initiator provides the only example for transcription initiation in the absence of a TBP. While the Inr element lies upstream from the TSS in the *muPARP-1* promoter, it is not immediately connected to any of the described YY1 sites, the closest being BM7. Although for the present study the precise functional assessment of BM7 was beyond reach, there is evidence to suggest that its strategic placement within the Kozak element next to the PARP-1 binding motif allows it to play a major role in *muPARP-1* regulation.

Many activation and repression models implicating YY1 have been proposed [40] and we cannot exclude that PARP-1 may become down- or upregulated in a context-dependent manner. Even if we restrict our considerations to the transcriptional level, YY1 is known for its multifunctional properties as it has been implicated in positive and negative regulation depending on the promoter [77], [78]. To explain the divergent functions of YY1, Fry and Farnham [79] put forward the hypothesis that the transcriptional activity of YY1 is influenced by its ability to bend DNA, and by physical interactions with a variety of basal and site-specific factors. Using well-defined synthetic promoters in which the YY1 binding site was inserted between the TATA box and the NF1 recognition sequences, these authors could show that the YY1 site stimulated promoter activity when placed between the NF1 binding site and the TATA box, but not when the positions of the YY1 and NF1 were switched. These and other results suggest that YY1-induced DNA bending *via* BM7 brings activators closer to the basal transcription complex and stimulates transcription while the association of YY1 with upstream elements (BM1, BM4) counteracts any YY1-mediated activation steps at BM7.

Our results highlight the ability of YY1 to associate with distal as well as proximal promoter regions and they point at context-dependent functions of *muPARP-1*. Protein-protein interactions have been related to the promoter context-dependent behaviour of YY1 in the human papilloma virus 18 (HPV18) upstream regulatory region, which provides multiple YY1 binding sites [80]. It was observed that for HeLa cells YY1 takes the role of an activator of HPV18 while in HepG2 cells it behaves as a repressor. In the first case, the promoter-proximal site serves as a positive regulatory element only when a ‘switch region’ is present 130 bp upstream from the YY1 site. A member of the C/EBP family of transcription factors, C/EBP*β*, binds the switch region and converts YY1 function from repression to activation [81]. Thus, the repressor activity of YY1 depends on protein-protein interactions with transcription factors at a nearby position and may explain why YY1 can activate some promoters while repressing others in the same cell. In addition to YY1-protein interactions, the dual transcriptional activities of YY1 are most likely affected by posttranslational modifications. A more recent explanation relies on interferences from a related protein, YY2, which reveals an overlapping spectrum of activities^37^. Regarding the complexity of the system and some operational restrictions that hamper the complete functional assessment of the proximal binding motif (BM7), the mechanistic basis of its activity will have to await further dedicated studies.

### Concluding Remarks

We established that the muPARP-1 gene core promoter is punctuated by three YY1 binding sites and distinct YY1 regulatory control points. While functional analyses have unequivocally shown that the two distal sites, BM1 and BM4, mediate negative effects on PARP-1 transcription, supporting the negative feedback loop of PARP-1, the precise role of the proximal high-affinity element BM7 remains to be fully uncovered, the more so as its composite nature may also enable positive transcriptional effects of YY1, in striking contrast to BM1 and BM4. There are indications that these YY1 binding motifs modulate promoter activity *via* a succession of concerted interactions. Thus, the most distal site BM1 lies adjacent (15 bp) to a PARP-1 binding consensus motif II where it flanks a DNA unpairing element (UE1) while the proximal BM7 is located next (3 bp) to the PARP-1 consensus sequence IV. Our data underline the versatility of YY1 switch functions by which *muPARP-1* promoter activity can be adapted to individual cellular requirements. YY1 thus emerges as an important component of the mechanism that oversees the maintenance of cellular homeostasis.

## Materials and Methods

### Mouse PARP-1 Gene Promoter

The sequence of the *muPARP-1* promoter (774 bp) spanning from positions −572 to +202 and predicted by GenomatixSoftwareGmbH (Munich) was described previously [31]. The core *muPARP-1* was searched for the presence of the YY1 core binding motifs ‘CCAT’ and ‘ACAT’, established as the most frequent YY1 core sequences in eukaryotic cells [50].

### Reporter Gene Constructs

Mouse genomic DNA was extracted from NIH3T3 cells and PCR-amplified following standard procedures [31]. In order to amplify the predicted *muPARP-1* promoter region, the following primers were used: upstream 5′-CATGGATCCCTGTGAGTTC-3′ and downstream 5′-GCGGAGGGAGTCCTTGGGAATACTC-3′ to yield a 774 bp product spanning a portion of the mouse PARP-1 5′regulatory region. The resulting amplification product was cloned into the pCR21 vector using the TA Cloning Kit (Invitrogen), sequenced, digested with HindIII and ClaI, and subcloned into the pSLGTKneo vector, a firefly luciferase/green fluorescent protein (GFP) gene expression vector optimized for the analysis of enhancer and promoter sequences. The obtained vector pPARPlucTKneo contains the muPARP-1 gene promoter fragment (positions −572 to +202) that drives the transcription of the luciferase gene. The vector was amplified in the chemically competent bacterial strain Top10F’ and subsequently used to transfect NIH3T3 cells. However, the pPARPlucTKneo reporter plasmid exhibited negligible activity in comparison with pSLGTKneo. Therefore, an extended construct, starting from position +100, was cloned. The *muPARP-1* promoter was PCR-amplified using mouse genomic DNA as a template and the following primers: upstream 5′-CTGCTCAATCAGGAATGATTCATAGACA-3′ and downstream 5′-TCCTTCTCGTGCTGCAGCGG-3′. The amplification product was cloned in the pMDICluc vector using SpeI and XhoI. The ampicillin gene served as a selection marker. This reporter plasmid or pPARPluc, contains the firefly luciferase reporter gene and the core *muPARP-1* promoter which is extended at its 5′ end by 384 bp and is slightly reduced at its 3′ end. Its total length is 1034 bp; it encompasses the region −956/+100 bp. This plasmid is fully functional. The *muPARP-1* promoter was divided into four fragments (designated as PARP-1 promoter fragments 1 to 4) that were amplified by PCR and cloned into pCR2.1 TOPO.

### Polymerase Chain Reaction (PCR)

PCR was used for the amplification of 200–250 bp fragments of the *muPARP-1* promoter which were subsequently cloned via TOPO TA cloning and for analyses of ChIP samples. The standard 20 µl PCR reaction consisted of the Expand Long Template PCR System DNA polymerase mix (Roche), 1x expand long template buffer 2, 250 µM dNTPs each, 1 µl HMW DNA as template and 20 pmol of each forward and reverse primer. After initial denaturation of the template DNA at 95°C for 5 min, 30 cycles of three subsequent steps were performed: denaturation for 5 min at 95°C, annealing for 30 s at 56–62°C; elongation for 2 min at 68°C; a final elongation at 68°C was conducted for 5 min to complete all ongoing elongation reactions.

### Rapid Cloning of Taq Polymerase Amplified PCR Products (TOPO TA Cloning®)

Cloning was performed as described in the manual of the TOPO TA Cloning® Kit (Invitrogen). Ligated vectors were introduced into *E.coli* DH10B or *E. coli* XL1-blue cells by electroporation.

### Cell Culture and Transient Transfection

NIH3T3 cells (ATCC, CRL-1658), derived from mouse embryonic fibroblasts and PARP-1 knock-out (PARP-1^−/−^) mouse fibroblasts (obtained from Valérie Schreiber, Département Intégrité du Génome UMR7175-LC1 CNRS, Ecole Supérieure de Biotechnologie de Strasbourg, Illkirch, France) were used. The cells were cultured in DME medium (Sigma) supplemented with 10% foetal calf serum (FCS), 1x glutamine, 100 U/mL penicillin, and 100 µg/mL streptomycin. The cells were grown at 37°C under 5% (v/v) CO_2_ and 90% humidity. The cells were counted using a Casy® cell counter (Innovatis). NIH3T3 cells were transfected using Lipofectamine™ 2000 (Invitrogen). The day before transfection, cells were plated in 24-well plates at 4×10^4^ cells/well, being 70% confluent after 24 h. The transfection was conducted according to the manufacturer’s instructions. After 2 h of growth, 0.5 ml of DMEM with 20% FCS was added to a final concentration of 10%. Transiently transfected cells were harvested two days after transfection for measuring the luciferase activity.

The functioning of the *muPARP-1* promoter was studied in NIH3T3 cells after transfection with the firefly luciferase gene expression vector described above, and in PARP^−/−^ cells over expressing YY1 as a result of cotransfection with vector pcDNA3.1FLAGYY1, a mammalian expression vector for FLAG-tagged human YY1 based on the vector pcDNA3.1 (Invitrogen) which contains the CMV promoter and a neomycin resistance gene (obtained from Dr. Martin Klar, Department of Neonatology, Campus Virchow-Klinikum, Charité-Universitätsmedizin, Berlin, Germany). A cDNA-based PARP-1 expression construct pECV PARP was introduced into PARP^−/−^ cells. PARP^−/−^ cells were transfected with reporter plasmids with mutations in two separate YY1 binding sites, designated as pPARPluc BM1^mut^ (which is based on pPARPluc but contains a mutation in YY1 binding site BM1 of the PARP-1 promoter), and pPARPluc BM4^mut^ (which contains a mutation in YY1 binding site BM4), respectively.

### Site Directed Mutagenesis

Base pairs of the YY1 core sequence within the BM7 region contained in the *muPARP-1* promoter were exchanged. The sequences of the five oligonucleotide probes with mutated base pairs (referred to in the text as BM7 m1 to m5), are presented in the legend to Fig. 5. Complementary primers carrying mutations were used in PCR with *PfuTurbo* DNA Polymerase (Stratagene). An initial denaturation (95°C, 5 min) was followed by 18 cycles with each cycle including denaturation for 30 s at 95°C, annealing for 1 min at 55° to 72°C and elongation for 7 min at 68°C. Following temperature cycling, the methylated and semi-methylated parental strands were selectively digested with DpnI (Stratagene). The mutation-containing vectors were used to transform *E. coli* TOP10 competent cells.

### Dual-Luciferase® Reporter Assay System

Cell lysates of transfected cells were prepared and measurements were conducted as described in the manual for the Dual-Luciferase® Reporter Assay System (Promega). Directly after measuring firefly luciferase activity, the enzymatic reaction of the latter is inhibited and the appropriate substrate and buffer conditions for *Renilla* luciferase were established by addition of the Stop&Glo®reagent. Firefly luciferase activity was normalized for total protein content by the bicinchoninic acid reaction (BCA assay).

### Cis-DDP Crosslinking and Chromatin Immunoprecipitation Experiments

The cis-DDP was used at 2 mM concentration as a crosslinking reagent. Chromatin immunoprecipitation (ChIP-IT® Express Chromatin Immunoprecipitation Kits; Actve Motif) was performed according to the manufacturer’s instructions.

### Preparation of Nuclear Extracts

Nuclear extracts served as a source of YY1 protein in EMSA. Nuclear extracts were prepared using the NucBuster™ Protein Extraction Kit (Novagen).

### Electrophoretic Mobility Shift Assay

The following probes were used in EMSA (described in detail in the Results section): the *muPARP-1* promoter fragment; oligonucleotides containing YY1 binding motifs (designated BM1 to 7); oligonucleotides containing mutations of the YY1 core sequence within BM7; PARP-1 promoter fragments 1 to 4. Oligonucleotides (30 bp) were labelled with γ-[^32^P]dATP (3000 Ci/mmol), using polynucleotide T4 kinase and the PARP-1 promoter fragments (200–270 bp) were labelled with α-[^32^P]dATP, using Klenow polymerase. The DNA was cleaned with G-50 columns (Amersham). NIH3T3 nuclear extracts (10 µg) were added in a buffer containing 12.5 mM Hepes (pH 7.9), 15 mM MgCl_2_, 0.5 mM EDTA, 50 mM KCl, 2 mM-mercaptoethanol, 0.05% (v/v) Nonidet P-40, and 7.5% glycerol. After incubation for 10 min at room temperature, the radioactively-labelled DNA fragments (about 200,000 cpm/µl) were added and incubation was carried out at 37°C for 30 min. Poly(dIdC) (2 µg) was used as a competitor DNA in each binding reaction. Competition reactions were performed in order to illustrate the specificity of the protein:DNA interactions. Each reaction contained a 200-fold molar excess of particular unlabeled oligonucleotides (BM 1–7) or *muPARP-1* promoter fragments 1–4. For super shift experiments, 1 µg of antibodies (anti-PARP-1 antibody; C2-10 (ALEXIS Biochemicals), and 1 µg of anti-human YY1 (H-414) rabbit polyclonal antibody (Santa Cruz Biotechnology)) were added to the protein mixture and incubated at room temperature for 30 min. Reaction mixtures were subjected to non-denaturing electrophoresis. For oligonucleotides, an 8% polyacrylamide gel in 0.25× Tris-borate-EDTA buffer (0.5× TBE) was used whereas the PARP-1 promoter fragments were run on a 5% gel at 140 V for 3.5 and 4.5 h, respectively. The dried gels were kept in phosphor screen-exposition cassettes for 1 to 3 days. The autoradiograms were analysed in a Phosphor-Imager using Image Quant ver. 5.0 (Molecular Dynamics) software.

Non-labelled *muPARP-1* promoter fragment (100 ng of 774 bp long fragment) and circular plasmid pPARPlucTKneo (in amount that 100 ng of promoter is present in the reaction mixture) were used in Fig. 2 in non-radioactive EMSA. Recombinant proteins, YY1 (Santa Cruz) and PARP-1 (Alexis), were used in the amount of 100 ng. In these experiments the formed nucleoprotein complexes were separated on 0.8% agarose gel in 1xTEA buffer.

### Protein Procedures

Proteins were quantified by the bicinchoninic acid (BCA) assay [82]. Absorption at 562 nm was measured with a Multiskan EXphotometer. Samples (20 µg) of proteins separated by SDS-polyacrylamide gel electrophoresis (PAGE) [83] were electro blotted onto a PVDF membrane. Immunoblot analysis was performed using mouse monoclonal anti-PARP-1 antibody (1∶10,000) (C2-10,Alexis), rabbit polyclonal anti-human YY1 (H-414) antibody (1∶10,000) (Santa Cruz Biotechnology) and rabbit monoclonal anti-mouse actin (Ab-1) antibody (1∶10,000) (Calbiochem). The blots were probed by horseradish peroxidase-conjugated secondary antibodies (Cell Signalling). Staining was performed by the chemiluminescent technique according to the manufacturer’s instructions (AmershamBiosciences).
